# Supplementary material for: HIVprotI: an integrated web based platform for prediction and design of HIV proteins inhibitors
Source: J Cheminform. 2018 Mar 9;10:12. doi: 10.1186/s13321-018-0266-y (PMC5845081; doi:10.1186/s13321-018-0266-y)
Supplement: Supplementary file 2 — Additional file 2. Source code of HIVProtI web server. [file 13321_2018_266_MOESM2_ESM.zip › HIVprotI_Source-code/hivprotI/weka-3-6-6/doc/weka/experiment/ResultListener.html]

ResultListener


---


|  |  |  |
| --- | --- | --- |
| **Overview** \| **Package** \| **Class** \| **Tree** \| **Deprecated** \| **Index** \| **Help** \| **Weka's home** | |  |
| **PREV CLASS**   **NEXT CLASS** | **FRAMES**    **NO FRAMES**     **All Classes** |  |
| SUMMARY: NESTED \| FIELD \| CONSTR \| METHOD | DETAIL: FIELD \| CONSTR \| METHOD |  |


---


## weka.experiment Interface ResultListener

**All Superinterfaces:**: java.io.Serializable

**All Known Implementing Classes:**: AveragingResultProducer, CSVResultListener, DatabaseResultListener, DatabaseResultProducer, InstancesResultListener, LearningRateResultProducer

---

``` public interface ResultListener extends java.io.Serializable ```

Interface for objects able to listen for results obtained
by a ResultProducer

**Version:**
:   $Revision: 1.7 $

**Author:**
:   Len Trigg (trigg@cs.waikato.ac.nz)

---

| **Method Summary** | |
| --- | --- |
| `void` | `acceptResult(ResultProducer rp, java.lang.Object[] key, java.lang.Object[] result)`             Accepts results from a ResultProducer. |
| `java.lang.String[]` | `determineColumnConstraints(ResultProducer rp)`             Determines if there are any constraints (imposed by the destination) on additional result columns to be produced by resultProducers. |
| `boolean` | `isResultRequired(ResultProducer rp, java.lang.Object[] key)`             Determines whether the results for a specified key must be generated. |
| `void` | `postProcess(ResultProducer rp)`             Perform any postprocessing. |
| `void` | `preProcess(ResultProducer rp)`             Prepare for the results to be received. |

**Method Detail**

### determineColumnConstraints

```
java.lang.String[] determineColumnConstraints(ResultProducer rp)
                                              throws java.lang.Exception
```

:   Determines if there are any constraints (imposed by the
    destination) on additional result columns to be produced by
    resultProducers. Null should be returned if there are NO
    constraints, otherwise a list of column names should be
    returned as an array of Strings.

    :   **Parameters:**: `rp` - the ResultProducer to which the constraints will apply **Returns:**: an array of column names to which resutltProducer's additional results will be restricted. **Throws:**: `java.lang.Exception` - if an error occurs

---


### preProcess

```
void preProcess(ResultProducer rp)
                throws java.lang.Exception
```

:   Prepare for the results to be received.

    :   **Parameters:**: `rp` - the ResultProducer that will generate the results **Throws:**: `java.lang.Exception` - if an error occurs during preprocessing.

---


### postProcess

```
void postProcess(ResultProducer rp)
                 throws java.lang.Exception
```

:   Perform any postprocessing. When this method is called, it indicates
    that no more results will be sent that need to be grouped together
    in any way.

    :   **Parameters:**: `rp` - the ResultProducer that generated the results **Throws:**: `java.lang.Exception` - if an error occurs

---


### acceptResult

```
void acceptResult(ResultProducer rp,
                  java.lang.Object[] key,
                  java.lang.Object[] result)
                  throws java.lang.Exception
```

:   Accepts results from a ResultProducer.

    :   **Parameters:**: `rp` - the ResultProducer that generated the results: `key` - an array of Objects (Strings or Doubles) that uniquely identify a result for a given ResultProducer with given compatibilityState: `result` - the results stored in an array. The objects stored in the array may be Strings, Doubles, or null (for the missing value). **Throws:**: `java.lang.Exception` - if the result could not be accepted.

---


### isResultRequired

```
boolean isResultRequired(ResultProducer rp,
                         java.lang.Object[] key)
                         throws java.lang.Exception
```

:   Determines whether the results for a specified key must be
    generated.

    :   **Parameters:**: `rp` - the ResultProducer wanting to generate the results: `key` - an array of Objects (Strings or Doubles) that uniquely identify a result for a given ResultProducer with given compatibilityState **Returns:**: true if the result should be generated **Throws:**: `java.lang.Exception` - if it could not be determined if the result is needed.


---


|  |  |  |
| --- | --- | --- |
| **Overview** \| **Package** \| **Class** \| **Tree** \| **Deprecated** \| **Index** \| **Help** \| **Weka's home** | |  |
| **PREV CLASS**   **NEXT CLASS** | **FRAMES**    **NO FRAMES**     **All Classes** |  |
| SUMMARY: NESTED \| FIELD \| CONSTR \| METHOD | DETAIL: FIELD \| CONSTR \| METHOD |  |


---
